# Symbolic Moral Self-Completion – Social Recognition of Prosocial Behavior Reduces Subsequent Moral Striving

**DOI:** 10.3389/fpsyg.2020.560188

**Published:** 2020-09-04

**Authors:** Moritz Susewind, Gari Walkowitz

**Affiliations:** ^1^Department of Child and Adolescent Psychiatry, Psychosomatics and Psychotherapy, Centre for Mental Health, University Hospital of Würzburg, Würzburg, Germany; ^2^Kitzberg Center for Psychosomatic Medicine and Psychotherapy, Bad Mergentheim, Germany; ^3^Digital Ethics Group, TUM School of Governance, Technical University of Munich and Center Digitization, Munich, Germany; ^4^National Research University Higher School of Economics, Moscow, Russia

**Keywords:** prosocial behavior, social influence, social recognition, self-regulation, moral balancing

## Abstract

According to theories on moral balancing, a prosocial act can decrease people’s motivation to engage in subsequent prosocial behavior, because people feel that they have already achieved a positive moral self-perception. However, there is also empirical evidence showing that people actually need to be recognized by others in order to establish and affirm their self-perception through their prosocial actions. Without social recognition, moral balancing could possibly fail. In this paper, we investigate in two laboratory experiments how social recognition of prosocial behavior influences subsequent moral striving. Building on self-completion theory, we hypothesize that social recognition of prosocial behavior (self-serving behavior) weakens (strengthens) subsequent moral striving. In Study 1, we show that a prosocial act leads to less subsequent helpfulness when it was socially recognized as compared to a situation without social recognition. Conversely, when a self-serving act is socially recognized, it encourages subsequent helpfulness. In Study 2, we replicate the effect of social recognition on moral striving in a more elaborated experimental setting and with a larger participant sample. We again find that a socially recognized prosocial act leads to less subsequent helpfulness compared to an unrecognized prosocial act. Our results shed new light on the boundary conditions of moral balancing effects and underscore the view that these effects can be conceptualized as a dynamic of self-completion.

## Introduction

### Prosocial Behavior Dynamics and Social Recognition

Imagine you consider donating money for a good cause. After making up your mind, you decide to give a generous amount to a non-profit organization and hand it over personally at their local office. Having left their building, you are approached by a person asking for support for another social initiative. Would you be willing to participate? According to theories on moral balancing (Effron and Monin, 2010; Mazar and Zhong, 2010), you probably wouldn’t, because your previous donation has provided you with a “license” - it allows you to derive a positive self-perception, implying that you have achieved or even exceeded your moral ideal (Wicklund and Gollwitzer, 1981). This, in turn, decreases your motivation to engage in subsequent good cause.

Now imagine that your generous donation has not been acknowledged by anyone, because you dropped it anonymously in the organization’s mailbox. Would you still feel licensed to turn down a subsequent request for help? Essentially, one could think that it makes no difference whether somebody has recognized your prior prosocial act. After all, you know what you have done, and this should be sufficient to assure yourself that you are of good character. However, there is empirical evidence showing that people actually need to be recognized by others in order to establish and affirm their self-perception through their actions (Wicklund and Gollwitzer, 1981; Gollwitzer, 1986; Gollwitzer et al., 2009). If others fail to recognize previous identity-building actions, people subsequently show consistent or even increased striving for similar actions.

In this paper, we investigate whether social recognition of prosocial acts indeed discourages future moral striving. After all, there is growing evidence that replications of balancing effects sometimes fail (Urban et al., 2019), which indicates that there are important moderators at work (Merritt et al., 2010; Mullen and Monin, 2016). Social recognition could be one of those moderators. We conduct two studies comprising controlled laboratory experiments where we examine participants’ real behavior and ensure an incentive compatibility of participants’ decisions. In our studies, we investigate the impact of prosocial or self-serving behavior, carried out in the first stage of the experiments, on subsequent helping behavior, measured in the second stage of the experiments. We define behavior as “prosocial” when it involves providing benefits for others, the environment, or society as a whole (cf. Schwartz, 1992, 2007). Accordingly, we define behavior as “self-serving,” when it is aimed at one’s own benefit neglecting the benefit of others. We conjecture that not being recognized for prior prosocial acts leads to consistent or even increased prosocial striving subsequently.

We derive our hypotheses from self-completion theory (Wicklund and Gollwitzer, 1981; Gollwitzer, 1986; Gollwitzer et al., 2009). This theory states that social recognition allows people to derive a sense of progress or goal attainment from their goal-congruent actions, which in turn influences future goal-striving. The potential influence of social recognition on moral balancing effects has been previously discussed (Monin and Miller, 2001; Merritt et al., 2010; Jordan et al., 2011; Lalot et al., 2019). Yet, to the best of our knowledge, it has not been empirically tested explicitly. Recently, Lalot et al. (2019) proposed a self-completion account to explain balancing effects. However, they mainly focused on minority vs. majority influence as a moderating variable, i.e., the normative influence resulting from alleged opinions presented to participants.

In our studies, we observe actual social recognition, i.e., the impact of being actually recognized and seen in one’s prosocial actions. So far, only Monin and Miller (2001) have observed actual social recognition. Yet, they did not include a group derived from social recognition.

If social recognition indeed influenced moral balancing, this would shed a new light on potential boundary conditions for moral balancing effects. It would also significantly contribute to the theoretical understanding of moral balancing, as Blanken et al. (2015, p. 540) have reiterated: “… the specific conditions under which moral licensing^1^ is likely to occur remain unclear.”

### Moral Balancing in the Context of Self-Completion Theory

People tend to define themselves as moral individuals and seek confirmation for this belief (Steele, 1988; Aquino and Reed, 2002; Dunning, 2007). Dunning (2007) argued that many decisions people take in their everyday lives are influenced by their general belief that they are of good character. The literature also shows that people appreciate situations in which they can cultivate their prosocial goals and self-perception (Monin and Miller, 2001; Khan and Dhar, 2006; Silverman et al., 2013).

Although prosocial behavior is generally perceived as positive, part of the literature shows that people do not behave consistently across situations. In particular, evidence conveys that, when past prosocial behavior reflects positively on people’s self-perception, people tend to expose less future prosocial striving (Monin and Miller, 2001; Khan and Dhar, 2006; Merritt et al., 2010). This phenomenon is termed *moral licensing* or *moral balancing*. For instance, Jordan et al. (2011) found that participants who recalled a prosocial action from their past showed fewer prosocial intentions afterward compared to a control group that recalled a neutral or an immoral action. In a similar vein, Mazar and Zhong (2010) found that buying green (vs. regular) products licensed people to be less generous subsequently. Taken together, this part of the literature suggests that there are different channels through which people can establish a positive self-perception that serves as a moral license. What appears to be important for moral balancing is that people’s past behavior is a symbolically meaningful indicator of prosociality.

Contrary to the above findings, other contributions convey that people act consistently. Their prosocial and, more generally, moral behavior, seems to be positively correlated. For example, Peysakhovich et al. (2014) found that positive transfers in the public-goods game, the dictator game, and in the trust game are positively correlated. Capraro et al. (2014) found that cooperation in the prisoner’s dilemma game and dictator-game giving are positively correlated. Biziou-van-Pol et al. (2015) found that lying aversion, cooperation in the prisoner’s dilemma, and dictator-game giving are positively correlated. Capraro and Rand (2018) and Tappin and Capraro (2018) found that moral choices in the trade-off game, cooperation in the prisoner’s dilemma, and dictator-game giving are positively correlated. Finally, in a recent study, Walkowitz (2020) shows that transfers in the dictator game are positively correlated with participants’ social value orientation [measured after the dictator game by the incentivized social values orientation slider measure from Murphy and Ackermann (2014)], and a donation to charity. These results seem not to be in line with moral balancing in general; but in the light of our theory, a potential explanation is that, in these experiments, participants’ prosocial behavior is not socially recognized, i.e., they take their prosocial decisions in the laboratory or online, unobserved and anonymously.

### The Importance of Social Recognition

Social recognition lies at the heart of symbolic self-completion theory (Wicklund and Gollwitzer, 1981; Gollwitzer, 1986; Gollwitzer et al., 2009). According to this theory, people conceptualize facets of their own identity as personal goals. People then use a wide array of activities that reflect on their personal goals to build their identity, a process called symbolic self-completion. For instance, people can exhibit behavior that reflects their identity goals in order to achieve a state of self-completion. Alternatively, they can display material symbols congruent with such goals or simply ascribe themselves goal-congruent characteristics. In either case, self-completion theory predicts that successfully enacting symbolic activities leads to fewer subsequent congruent activities, because people derive a sense of progress or goal achievement from successful symbolic activities, which in turn leads to reduced tension and striving for similar goal-congruent activities (Wicklund and Gollwitzer, 1981). On the other hand, if people fail to enact goal-relevant symbolic activities or act goal-incongruently, they experience a lack of progress and subsequently show increased striving for goal-congruent activities. Given these predictions, it does not come as a surprise that the theoretical mechanism outlined by self-completion theory has been used to explain moral balancing effects (Jordan et al., 2011).

Yet, the core predictions of self-completion theory have not been sufficiently addressed in the moral balancing literature. One central postulate states that people can only derive a sense of goal progress from their symbolic activities when these activities are recognized by others. Essentially, when people are not recognized by others, they lack a sense of progress because what they did “did not become a social fact” (Gollwitzer, 1986, p. 144). The reason for this is that, according to the theory, identity construction is always situated in a social context and can only emerge in relation to others. Thus, the symbolic impact of a given activity on an individual’s self-construction depends on the activity being recognized by others. This is why people continue striving for an identity goal if their previous activity remains unrecognized. It is important to note that the process described by self-completion theory has little to do with impression management, i.e., self-completion effects are not driven by strategic concerns to impress others (Gollwitzer, 1986). Consequently, for self-completion to occur, the mere presence of any audience is more important than its actual nature (Wicklund and Gollwitzer, 1981; Gollwitzer, 1986). In support of these assumptions, Gollwitzer (1986) present experimental evidence that self-relevant activities recognized by various audiences can discourage subsequent goal-striving. Specifically, one study showed that women who want to become mothers exhibited weaker striving to express their good mother skills when another participant had already noticed their skills before (Gollwitzer, 1986, Study 1). In a different series of studies, it was shown that university students performed weaker on tasks relevant to their study subject if the experimenter had recognized their performance intentions beforehand (Gollwitzer et al., 2009).

### Previous Research on Moral Balancing Relevant to Self-Completion Theory

Despite the fact that self-completion theory clearly points to the potential influence of social recognition on moral balancing effects, empirical research on this issue is scarce.

Longoni et al. (2014) show that positive vs. negative feedback on green shopping decisions influences people’s subsequent recycling behavior. However, despite using self-completion theory as a theoretical background, the authors do not discuss or investigate social recognition, which is a core feature of self-completion theory. In a similar vein, Lalot et al. (2019) propose self-completion theory as a framework, investigating the influence of alleged minority vs. majority opinion and pro-environmental vs. anti-environmental attitudes on moral balancing. They report the results of three online questionnaire studies and one classroom questionnaire study, which suggest overall that the participants’ score on a pro-environmental intentions scale was reduced (increased) by previously provided information about an alleged majority (minority) opinion in association with a previous assessment of the participants’ own pro-environmental behavior. While these results are in line with self-completion theory, once again the observed concepts are not comparable to actual social recognition.

Jordan et al. (2011, p. 10) discuss the possibility that moral balancing effects might be influenced by social recognition, but finally conclude that “private reflections on one’s past behavior may be enough to produce … compensatory effects.” However, the authors also state that their data do not explicitly allow them to test this assumption, acknowledging that further research is needed. In a similar vein, Monin and Miller (2001, p. 39) argue that “it is not critical that others know of one’s credentials for them to have a licensing (balancing) effect.” But the authors’ conclusion was only meant to imply that moral balancing effects are not driven by the participants’ self-presentational concerns. In support of this assumption, they ran an experiment designed to test whether people show moral balancing, because they want to impress the experimenter. Specifically, Monin and Miller (2001) compared two experimental conditions. In one experimental condition, the participants’ past prosocial behavior was witnessed by one experimenter, but the dependent variable measuring prosocial intentions was witnessed by a different experimenter. In a second experimental condition, the participants’ past prosocial behavior and subsequent intentions were witnessed by the same experimenter. The authors found that in both experimental conditions participants exhibited a moral balancing effect, showing lower prosocial intentions compared to a control condition that did not engage in previous prosocial behavior. This implies that the moral balancing effect was not based on strategic efforts to impress one experimenter. However, it does not imply that social recognition did not play a role. After all, participant behavior in both experimental conditions was recognized by *some* audience. Self-completion theory explicitly argues that the nature of the audience does not matter much as long as there is any audience present. In order to exclude the influence of social recognition, an experiment in which the participants’ behavior in one group was clearly not recognized by anyone is needed. To the best of our knowledge, no such experiment has been conducted so far (see also Blanken et al., 2015, for a recent review).

### Hypotheses of the Current Research

In order to provide a compelling test of the hypotheses derived from self-completion theory, we designed two studies examining participants’ real behavior as a dependent variable. The first experiment uses an elaborated social-recognition manipulation, typically used in self-completion studies, and analyzes the impact of prosocial and self-serving acts. The participants’ behavior is either observed by another person or carried out in private. The second experiment replicates the social recognition manipulation of the first experiment and focuses on the impact of a prosocial act in a more elaborated experimental design with a larger participant sample.

According to self-completion theory, the influence of social recognition can be tested by observing the influence of goal attainment and goal failure (Wicklund and Gollwitzer, 1981; Gollwitzer, 1986; Gollwitzer et al., 2009). Specifically, the test is to observe goal-congruent behavior under conditions with and without social recognition. Applied to our topic, this means testing whether people who engage in a prosocial task show less (goal-congruent) prosocial striving when their prosocial behavior was socially recognized vs. when it was not (Hypothesis 1, effect of prosocial goal attainment vs. failure). At the same time, we expect to observe that people who engage in a self-serving task show more prosocial striving when their self-serving behavior was socially recognized vs. when it was not (Hypothesis 2, effect of self-serving goal attainment vs. failure). After all, social recognition allows one to derive a feeling of goal attainment in either prosocial or self-serving goals. Without social recognition, the opportunity to engage in prosocial as compared to self-serving behavior might activate corresponding goals, but people do not experience a sense of goal progress or self-completion. They rather experience a sense of goal failure. Their goals remain active and stimulate goal-congruent striving in subsequent situations. In Study 1, we test both Hypotheses 1 and 2 in a single paradigm. As we expect opposing patterns in prosocial as compared to self-serving conditions, we test for an interaction effect.

## Study 1

### Participants

We recruited 180 students via invitation from a mailing list in a large German university (50.56% female, mean age: 22.96 years, *SD* = 3.07) to participate in a laboratory experiment. We included all participants who responded to our invitation. We did not increase our sample size further after a preliminary data analysis. We did not explicitly preselect participants based on commitment toward prosocial or self-serving goals. In line with other authors, we assume that the goal to act as a prosocial individual (Aquino and Reed, 2002; Dunning, 2007) and the goal to act as a self-serving individual (Dunning et al., 1991) are generic motives that people generally have.^2^ We report all measures, conditions, and exclusions. Participants were randomly assigned to one of four conditions in a 2 (recognition: with social recognition, without social recognition) × 2 (endorsement: prosocial, self-serving) design. Participants received a fixed amount of 3 € as compensation for attendance. Debriefing information was provided through the department’s homepage.

### Procedure

Table 1 provides an overview of the different stages of the experiment. Upon arrival in the laboratory, participants were randomly assigned to individual opaque cubicles. First, participants were invited to participate in an endorsement task. This task served as the prosocial versus self-serving behavior condition, i.e., the two conditions of our first independent variable. Specifically, we truthfully informed the participants that we assisted the student association of the university in collecting ideas about how to improve the environment of the students and their everyday lives and how future resources should be directed. To this end, we were running an opinion poll in which each participant was asked to choose six measures that he or she considered important and wanted to endorse. Following the logic of the task applied by Mazar and Zhong (2010), participants in the prosocial condition received a ballot sheet containing nine prosocial and three self-serving measures. Participants in the self-serving condition received a ballot sheet containing nine self-serving and three prosocial measures. The measures were selected after a pretest (*N* = 41), in which 30 different potential engagements were tested with regard to perceived prosociality and perceived selfishness. For example, prosocial items included in the main study were “I endorse that public transport is improved such that people with disabilities can reach university much easier and in shorter time” or “I endorse that the public cafeteria supplies specific healthy food for people suffering from allergic conditions.” The items on selfishness were basically the same in content, but phrased in a self-serving way. For instance, in the self-serving condition, an item would read: “I endorse that public transport is improved such that my personal way to reach university (fill in location: ____) is much easier and shorter in time.” Thus, only the framing of the statements, but not their actual content, differed across conditions. Participants were asked to mark the items they wanted to endorse and to add their suggestions. Although this task is not incentivized monetarily, incentive compatibility was given. Participants acted out real behavior resulting in potential future consequences for themselves and others. We truthfully informed participants that the results of their opinion poll would be forwarded and processed by the universities’ student associations and could result in measures taken in the interest of the students.

**TABLE 1:** Study 1: Overview on the experimental stages.

First stage		Second stage	
1. Elicitation of prosocial or self-serving behavior	2. Social recognition	3. Introduction to the second part of the experiment, questionnaires	4. Elicitation of subsequent prosocial behavior
Measure: Poll where participants were asked about their ideas on how to improve the environment of students and the students’ everyday lives, and how future resources should be directed. Included prosocial and self-serving items. Incentives: Participants’ suggestions were taken into account by the university administration; fixed amount for participation.	With social recognition: Public (with regard to the experimenter) handover and receipt of the poll. Without social recognition: Private handover of the poll via a sealed envelope being put into a box.	Filler tasks, Manipulation checks, Socio-economic questionnaire.	Willingness to help the experimenters in developing materials necessary for a different experimental study. Measure: Staying longer in the laboratory, solving computational puzzles, and evaluating their difficulty. Incentives: Participants had to invest their time in order to support the experimenters.

Second, following a procedure inspired by Gollwitzer et al. (2009), we implemented our second independent variable “social recognition.” Participants in the conditions with social recognition were asked individually to hand over their poll sheet and booklet to the experimenter in an adjacent room after they had made their choices. The experimenter read out loud the endorsed options to confirm that the sheet had been filled out properly. Participants in the conditions without social recognition were asked to take out the poll sheet from their booklet, place it into a provided envelope, seal the envelope, and drop it into a black box located next to the laboratory door. Participants were truthfully told that their suggestions would not be handled by the experimenters, but anonymously by external personnel.

Third, we introduced participants to the second part of the experiment, containing filler tasks and the main dependent variable (actual helping behavior, see below). Participants indicated their preferences for several pictures showing leisure activities and filled out a questionnaire on different questions concerning how they spend their leisure time.

Afterward, participants were told that the study was almost over. We then informed them that we needed volunteers to help us out with an additional task *after* the current experiment. Specifically, participants were asked if they were willing to help us develop materials necessary for a different study on “performance” (see Twenge et al., 2007; Gino and Desai, 2012, for similar tasks). The task involved solving computational puzzles and evaluating their difficulty. Each puzzle was solved by finding two numbers adding up to exactly 10 in a table of 12 numbers. Participants were provided an example of a puzzle in their booklet, and they were told that they could solve and evaluate up to 50 puzzles. It was emphasized that their extra work was beyond the time scope of the current experiment, i.e., participants had to invest extra time in order to help the experimenters. Further, they were assured that they would be compensated for the current experiment, regardless of whether they engaged in any additional work or not - as announced at the beginning of the experiment.

Finally, before participants could start to work on the puzzles, we asked them to complete a short final questionnaire. The questionnaire contained manipulation checks for our conditions and socio-demographic questions. We asked people to think back to the endorsement task of the experiment and evaluate on six 7-point scales whether they had experienced themselves and their behavior in a positive way (example items: “I experienced myself as a person who has many positive characteristics,” “I experienced my behavior as something that is useful for disadvantaged people”). The six items were aggregated to a prosociality score (Cronbach’s α = 0.75, the last two items were reversed). The prosociality score was used to check whether the behavior in the prosocial conditions was perceived as more prosocial than the behavior in the self-serving conditions. We also asked the participants two questions concerning the perceived social recognition of their decisions: “In your opinion, did the experimenter notice what measures you endorsed?”, “In your opinion, was the endorsement task conducted anonymously?” (Cronbach’s α = 0.78). Last, sociodemographic information was collected. Our dependent measure was the number of matrices solved after the end of the experiment. Participants who wanted to leave were individually picked up by the experimenters from their cubicles and accompanied to a separate room for their payoff.

### Results

Three participants terminated the experiment before completing the main dependent variable, i.e., the matrix task. Due to their missing data, they were not included in the main analysis. One participant did not report age and gender, another participant did not provide manipulation checks. These participants were included in the main analysis and only omitted from analyses concerned by missing data.

#### Manipulation Checks

The experimental manipulations we applied were successful. Participants in the prosocial conditions (*M* = 4.0, *SD* = 1.0, 95% CI: 3.8–4.2) perceived their behavior as more prosocial than participants in the self-serving conditions [*M* = 3.5, *SD* = 0.9, 95% CI: 3.3–3.7; *t*(174) = 3.4, *p* < 0.001, *d* = −0.51]. Participants in the conditions with social recognition perceived their behavior to be recognized to a higher extent (*M* = 3.4, *SD* = 1.9, 95% CI: 2.9–3.8) than participants in the conditions without social recognition [*M* = 0.6, *SD* = 1.1, 95% CI: 0.3–0.8; *t*(174) = 11.8, *p* < 0.01, *d* = −1.80].

#### Subsequent Helping Behavior

**FIGURE 1:**
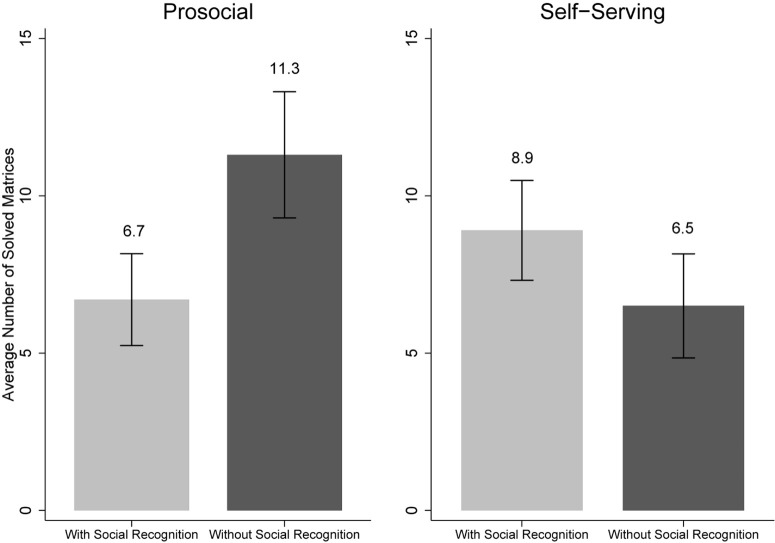
Main results of Study 1: mean number of matrices solved by recognition (with social recognition vs. without social recognition) and endorsement (prosocial vs. self-serving). The error bars reflect standard errors.

In order to test our main assumption that social recognition of prosocial behavior reduces subsequent helpfulness, while social recognition of self-serving behavior increases subsequent helpfulness, we tested the interaction effect of recognition (with social recognition, without social recognition) and endorsement (prosocial, self-serving) on the number of matrices solved as dependent variable (see Table 2 for descriptive statistics and Figure 1). A Kolmogorov–Smirnov test of normal distribution showed that our dependent variable did not achieve sufficient distribution for parametric testing [*KS*(177) = 0.2, *p* < 0.001]. Because the assumption of normality was violated, we used a non-parametric adjusted rank-transformed ANOVA (ART; Leys and Schumann, 2010) to test the interaction and main effects. The ART method basically performs an ANOVA analysis on a rank-scaled variable. Specifically, in step one, it transforms the dependent variable into a rank-scaled variable. In step two, it performs ANOVA testing on this variable. ART has been applied in a variety of publications across different research fields (see Schumann et al., 2014; Saarenheimo et al., 2016; Barbot and Carrasco, 2018, for examples) and is one of a few methods allowing for non-parametric testing of interaction.

**TABLE 2:** Study 1: Number of matrices solved by recognition (with social recognition vs. without social recognition) and endorsement (prosocial vs. self-serving).

	With social recognition		Without social recognition	
Endorsement	*M (SD)*	*N*	*M (SD)*	*N*
Prosocial	6.7 (9.8)	45	11.3 (13)	42
Self-serving	8.9 (10.5)	44	6.5 (11.2)	46

*Means (standard deviations), for number of matrices solved. N shows cell size.*

Based on the adjusted rank scores, we found no main effects of endorsement [*F*(1,174) = 0.4, *p* = 0.72, η^2^ = 0.001] or recognition [*F*(1,173) = 0.1, *p* = 0.72, η^2^ = 0.002]. However, in line with our assumptions, we found a significant interaction effect between endorsement and recognition [*F*(1,173) = 6.5, *p* = 0.01, η^2^ = 0.04]. A parametric univariate ANOVA resulted in the exact same pattern (no main effects, significant interaction effect).

To study the pattern of the interaction in detail, we used the non-parametric Mann–Whitney *U* test. Specifically, to test the effect of prosocial goal attainment vs. failure (Hypothesis 1), we calculated comparisons across the prosocial conditions. In the conditions where participants endorsed prosocial measures, they solved significantly fewer matrices (*M* = 6.7, *SD* = 9.8, 95% CI: 3.8–9.7) with social recognition than without social recognition [*M* = 11.3, *SD* = 13, 95% CI: 7.3–15.4; *U*(87) = 2.0, *p* = 0.05, η^2^ = 0.04]. Conversely, in the conditions where participants endorsed self-serving measures, they solved more matrices with social recognition (*M* = 8.9, *SD* = 10.5, 95% CI: 5.7–12.1) than without social recognition [*M* = 6.5, *SD* = 11.2, 95% CI: 3.2–9.9; *U*(90) = 1.9, *p* = 0.06, η^2^ = 0.03]. The difference was only marginally significant, however.

To complement the analysis, we also ran comparison tests across social recognition conditions. In the conditions with social recognition, participants solved fewer matrices after endorsing prosocial measures (*M* = 6.7, *SD* = 9.8, 95% CI: 3.8–9.7) than after endorsing self-serving measures [*M* = 8.9, *SD* = 10.5, 95% CI: 5.7–12.1; *U*(90) = 1.5, *p* = 0.12, η^2^ = 0.02], but the difference was not statistically significant. In the conditions without social recognition, participants solved significantly more matrices after endorsing prosocial measures (*M* = 11.3, *SD* = 13, 95% CI: 7.3–15.4) compared to self-serving measures [*M* = 6.5, *SD* = 11.2, 95% CI: 3.2–9.9; *U*(88) = 2.2, *p* = 0.03, η^2^ = 0.04].

In Study 1, we found support for Hypothesis 1 that people who engaged in a prosocial act exposed less goal-congruent prosocial striving when their behavior was socially recognized as compared to when it was not. We also found some support for Hypothesis 2 that people who engaged in a self-serving act were encouraged to continue prosocial striving when their behavior was socially recognized, as compared to when it was not. In addition, we found a specific influence of depriving people of social recognition. Without social recognition, the difference between the prosocial and self-serving conditions was also significant. Our results seem at odds with the assumption of Jordan et al. (2011, p. 10) that “private reflections on one’s past behavior may be enough to produce … compensatory effects.” Our findings suggest that there is a significant influence of social recognition consistent with self-completion theory.

In sum, Study 1 has some shortcomings. First, the sample size in Study 1 could have been too small, i.e., a replication is warranted to secure sustainability of effects. Also, despite our efforts with a pre-test, our manipulation check revealed that the prosocial manipulation was successful, but relatively subtle. Finally, we observed a restricted variance in our dependent variable due to the (previously announced) placement the helping task after the official end of the laboratory session. It was more demanding for participants to act prosocially as compared to self-servingly, because helping implied staying in the laboratory beyond the time scope initially announced. To address these issues and improve the design further, we designed a second experiment. We decided to focus on replicating the test of Hypothesis 1 (effect of prosocial goal attainment vs. failure), because it is more relevant to our assumptions.

## Study 2

In Study 2, we tested the effect of prosocial goal attainment vs. failure in a more elaborate experimental setting with a larger sample size. We developed an experimental setting that included a more direct condition of prosocial behavior that was also incentivized monetarily. We also chose a different time frame for the experimental sessions to prevent a skewed distribution of helping behavior at the second stage of the experiment. Given the effect size observed in Study 1, we assumed an effect of *d* = 0.40 to 0.50 to be plausible. A G-power analysis to calculate the necessary sample size, assuming a power of 0.90, yielded an *N* of 146 (cell size corresponding to 73).

### Participants

In total, we recruited 125 participants in our laboratory experiment (51.2% female, mean age = 25.7 years,^3^ *SD* = 7.3). Thus, we did improve statistical power significantly, though not entirely to the level aspired. Again, we included all participants who responded to our invitation. Participants were contacted via e-mail, and for this we availed of the department’s pre-registered subject pool. We did not increase our sample size further after a preliminary data analysis. We report all measures, conditions, and exclusions. During the experiment, participants were seated in individual and opaque cubicles. Participants were compensated with a fixed amount of 2.50 € along with the amount that they additionally earned in the experimental task described in the following.

### Procedure

Table 3 provides an overview of the different stages of the experiment. Upon arrival, participants received written instructions for the first decision task which served as prosocial behavior induction. In the task, participants had to decide upon a payoff for themselves and a donation to a UNICEF program for treating children with malaria. Participants had two options, A and B. If they decided to take option A, they received a payoff of 5.10 €. In this case, 0 € was donated to the UNICEF program. If participants decided to take option B, they received a payoff of 4.90 €. In this case, an amount of 2 € was donated to the UNICEF program by the participant. The donation was not subtracted from the participants’ payoff, but covered by the experimenters. Because the option B in the task was (a) more prosocial, (b) efficient, and (c) the cost for choosing option B was very low (0.20 €) as compared to an additional benefit of 2 € for the UNICEF program, we expected most participants to choose option B, i.e., to act prosocially. The sum of participant donations was calculated and transferred to UNICEF after the experiment by the experimenters. Participants later had the opportunity to verify this donation by a donation receipt stored at the experimenters’ office.

**TABLE 3:** Study 2: Overview on the experimental stages.

First stage		Second stage	
1. Elicitation of prosocial behavior	2. Social recognition	3. Introduction to the second part of the experiment, questionnaires	4. Elicitation of subsequent prosocial behavior
Measure: Choice of the higher donation to charity (Unicef). Incentives: Participants’ payoff dependent on their donation decision; fixed amount for participation.	With social recognition: Public (with regard to the experimenter) handover and receipt of the decision sheet. Without social recognition: Private handover of the decision sheet via a sealed envelope being put into a box.	Filler tasks, Manipulation checks, Socio-economic questionnaire.	Willingness to help the experimenters in developing materials necessary for a different experimental study. Measure: Staying longer in the laboratory, solving computational puzzles, and evaluating their difficulty. Incentives: Participants had to invest their time in order to support the experimenters.

After participants had marked their choice, they were randomly assigned to one of two experimental conditions serving as independent variable. Following a similar protocol as in Study 1, in the condition without social recognition, participants were instructed by the experimenter to put their decision sheet into an envelope, seal it, and drop it individually into a box. It was emphasized that sealed envelopes would be passed on and payouts would be organized absolutely anonymously at the end of the experiment. In the condition with social recognition, the experimenter approached the participants individually and recorded their decisions on a sheet of paper in their presence. Then, the participants’ sheets were folded and sealed, followed by the same payout procedure as described above.

Next, the experimenters administered two consecutive questionnaires containing dependent variables, e.g., manipulation checks for goal progress and social recognition. The first questionnaire contained a short description of 15 Likert-scale goal ratings (0 = I have made no progress; 6 = I have made considerable progress) on goals which could have potentially influenced the participants’ decisions in the previous decision task (prosocial goals and self-enhancement goals). Though we did not manipulate prosociality as in Study 1, we sought to control whether the donation task was associated with prosocial goals, e.g., induced prosociality. As hypothesized *a priori*, a principal component factor analysis (oblique rotation) revealed a two-factor solution according to the Guttmann–Kaiser criterion (factor loadings > 0.55). Eight items loaded on a factor we labeled “prosocial” (examples: being helpful, being fair; Cronbach’s α = 0.93). Seven items loaded on the second factor we labeled “self-enhancement” (examples: being ambitious, being goal-oriented, being adventurous; Cronbach’s α = 0.92). The second questionnaire contained three Likert-scale items (1 = not true at all; 7 = absolutely true) to assess whether the perceived social recognition (“The experimenter will be able to recall individual decisions after the experiment”; “I think the experimenter noticed which decision I took”; “I think my decision was anonymous”). The items (with the last item reversed) were aggregated to a social recognition scale (Cronbach’s α = 0.79).

Finally, we asked the participants again to help out with an additional task which, as in Study 1, served as our main dependent variable. As in Study 1, we asked them to help us develop study materials necessary for a subsequent study on performance. Materials and instructions were identical to the materials used in our first study. In contrast to Study 1, we administered the helping task about 30 min before the previously expected end of the experimental session. Participants had been informed about this time frame before. We intended that all participants could consider helping us, avoiding participant dropout because of external reasons (e.g., lecture, appointment, etc.).

Once participants had finished, experimenters in a separate payout room executed the individual and anonymous payouts, where participants, behind a curtain, could take for themselves one of two envelopes labeled either with “5.10 €” or “4.90 €.” The label and the content of the envelopes corresponded to the two possible payoffs resulting from the first decision task, including also the 2.50 € show-up fee.

### Results

From 125 participants, 95.97% made the donation to UNICEF in the first stage of the experiment. One participant could not be analyzed, because he/she terminated the experiment before completing the donation task. This participant was not included in the main analysis. Five participants (four in the condition without social recognition; one in the condition with social recognition) either decided not to donate to UNICEF (three participants) or made an ambiguous choice (two participants).^4^

#### Manipulation Checks

Confirming the validity of our donation task, participants across both conditions reported significantly more progress on prosocial goals (*M* = 3.2, *SD* = 1.5, 95% CI: 2.9–3.4) compared to self-enhancement goals [*M* = 1.1, *SD* = 1.3; *t*(122) = 15.7, *p* < 0.001, *d* = −1.45]. In line with the social recognition manipulation, participants in the condition with social recognition perceived higher social recognition (*M* = 4.1, *SD* = 1.9, 95% CI: 3.7–4.6) than participants in the condition without social recognition [*M* = 2.1, *SD* = 1.2, 95% CI: 1.7–2.4; *t*(122) = 7.5, *p* < 0.001, *d* = −1.34].

#### Subsequent Helping Behavior

For our main analyses (Hypothesis 1), we first analyzed the difference between the recognized and the unrecognized condition. Besides our efforts, the assumption of normality was again violated, [*KS*(124) = 0.2, *p* < 0.001], although the distribution seemed improved compared to Study 1. Accordingly, we again used a non-parametric Mann–Whitney *U* test to analyze the results.

**FIGURE 2:**
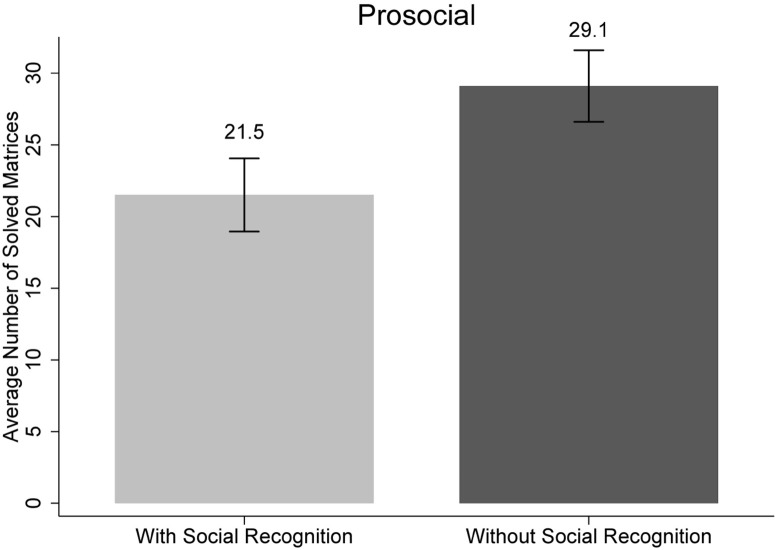
Main results of Study 2: mean number of matrices solved by recognition (with social recognition vs. without social recognition). The error bars reflect standard errors.

The test showed that participants in the condition with social recognition (*M* = 21.5, *SD* = 19.6, 95% CI: 16.4–26.6) solved significantly fewer matrices as compared to the participants who were in the condition without social recognition [*M* = 29.1, *SD* = 20.1, 95% CI: 24.1–34.1; *U*(124) = 2.0, *p* = 0.044, η^2^ = 0.03] (see Table 4 and Figure 2).

**TABLE 4:** Study 2: Number of matrices solved by recognition (with social recognition vs. without social recognition).

	With social recognition		Without social recognition	
Endorsement	*M (SD)*	*N*	*M (SD)*	*N*
Prosocial	21.5 (19.6)	59	29.1 (20.1)	65

*Means (standard deviations), for number of matrices solved. N shows cell size.*

In Study 2, we again found empirical support for our first hypothesis that social recognition induces goal attainment which, in turn, reduces subsequent helpfulness. Specifically, we found this effect in a study design where the independent variable was monetarily incentivized and the dependent variable was again operationalized as actual behavior. Further support for our main finding can be derived from an analysis where we pool the data from the two prosocial behavior conditions in Study 1 (with and without social recognition) with the data from the two prosocial behavior conditions in Study 2 (with and without social recognition). Although the operationalized prosocial behavior is different across the two studies, the dependent variable is structurally identical (number of correctly solved matrices). Testing the pooled data yielded that participants in the condition with social recognition solved significantly fewer matrices (*M* = 15.1, *SD* = 17.6, 95% CI: 11.7–18.5) as compared to the participants who were in the condition without social recognition [*M* = 22.1, *SD* = 19.7, 95% CI: 18.4–25.9; *U*(209) = 2.7, *p* = 0.01, η^2^ = 0.04].

## Discussion

### Significance and Theoretical Contribution of the Main Results

In the current article, we investigated the influence of social recognition on prosocial behavior dynamics. In Study 1, we found that a prosocial act leads to significantly less subsequent helpfulness when it was socially recognized as compared to not. This result was replicated in Study 2 with an improved study design and an elevated sample size. Also in Study 1, we found that people who engaged in a self-serving task were encouraged to continue with prosocial striving when their behavior was socially recognized as compared to not, although the socially recognized condition was only marginally significantly higher. Finally, we found a significant influence of depriving people of social recognition. Without social recognition, a prosocial task leads to more goal-congruent prosocial striving, while a self-serving task leads to less prosocial striving.

Overall, our results are in line with self-completion theory (Wicklund and Gollwitzer, 1981; Gollwitzer, 1986; Gollwitzer et al., 2009) and establish the significance of social recognition in the field of moral balancing effects. Some aspects of our results go beyond the scope of self-completion theory. In general, moral balancing effects have been observed across a wide range of different contexts and paradigms (Merritt et al., 2010; Blanken et al., 2015; Nilsson et al., 2017). There are different theoretical approaches to explain moral balancing effects. However, only self-completion theory can account for the unique influence of social recognition. In our research, we specifically observed that social recognition of prosocial acts reduces prosocial striving, while without social recognition prosocial striving is encouraged. This makes sense under the assumption that people can only derive a sense of progress or personal goal attainment from their symbolic activities when these activities are recognized by others. Given that self-completion theory is one of the most established theories relating to moral balancing, it is astonishing that its basic predictions have barely been investigated in this context before. While most balancing paradigms cannot disentangle if compensation for goal-failure and balancing after goal-achievement may account for the observed effect (the “donut problem”; Mullen and Monin, 2016), our data allowed for some further analysis. While we do not solve the donut problem, we were able to distinguish between the effect of social recognition dependent goal attainment (achieving prosocial standards) and goal failure (not achieving prosocial standards). In Study 1, we even observed a difference across conditions without social recognition. Thus, it seems that the effect of goal failure might even be stronger than the effect of goal attainment. This seems in line with Mullen and Monin’s (2016) observation that “compensation,” as they call it, is easier to observe than “licensing.” It is also congruent with the more general observation that adverse or unfavorable events exert stronger influence than pleasant or favorable ones (Rozin and Royzman, 2001). However, the effect of goal attainment clearly exists and was shown both in Study 1 and 2.

Although our results can be best explained by self-completion theory, they do not necessarily contradict alternative models of moral balancing. Self-regulation theory (Koo and Fishbach, 2008; Fishbach et al., 2009) proposes a similar mechanism to self-completion theory - perceived goal progress weakens subsequent goal-striving. This makes self-regulation theory compatible with self-completion theory and can partly explain our findings. However, self-regulation theory does not make predictions about the influence of social recognition. Therefore, it cannot fully explain our data. Further, self-regulation theory predicts that commitment to relevant goals promotes the opposite dynamic of moral balancing, i.e., moral highlighting. This prediction seems at odds with Gollwitzer’s predictions and with our results. A possible explanation for this discrepancy could be that the two theories define commitment differently. In fact, self-completion theory suggests that commitment is a situation-invariant attitude, i.e., a trait-like concept. In his studies, Gollwitzer often operationalized commitment as a personal characteristic or pre-existing role that someone possesses, such as being a parent, an athlete, or a student (Gollwitzer et al., 1982, 2009; Gollwitzer, 1986). We rely on the assumption that people are committed to their belief that they are of good character (Steele, 1988; Aquino and Reed, 2002; Dunning, 2007). On the other hand, in Fishbach’s studies, commitment is often operationalized as a situational state, referring to a specific action: it is operationalized as a cognition that is experimentally activated (Koo and Fishbach, 2008; Fishbach et al., 2009). For example, participants are primed with the commitment to keep in shape by answering questions about their “health and fitness” behavior (Fishbach et al., 2006). While pre-existing attitudes (self-completion theory) seem to be a precondition for moral balancing, situational primes of action-specific commitment (self-regulation theory) seem to prevent it. The latter effect could be caused by cognitive dissonance (Festinger and Carlsmith, 1959) resulting from the discrepancy of an individual’s behavioral history and the experimentally primed cognition. It could also relate to the mechanism described by self-perception theory (Bem, 1972), i.e., that people come to know their own attitudes by inferring them from observation of their own behavior. Keeping in mind this important difference calls for a more specific language and use of psychological concepts. In our framework, we relied on commitment as a pre-existing attitude which people hold about themselves, i.e., their general belief that they are of good character (Steele, 1988; Aquino and Reed, 2002; Dunning, 2007). Hence, our results are compatible with Gollwitzer’s concept of commitment.

Our results contribute to the moral balancing literature, because they help us to understand the boundary conditions of moral balancing effects. They also offer new insights into the nature of these effects. This is particularly necessary, as Blanken et al. (2015) concluded that none of the currently discussed moderators of moral balancing (free versus forced choice of good behavior; high vs. low rationalizability of cheating; recalling recent versus distant good behavior; having an outcome-based versus a rule-based mindset; focusing on goal progress vs. goal commitment; having no external incentive vs. having an external incentive for one’s moral behavior) significantly moderated the balancing effect in their meta-analysis.

Based on our results, one factor that possibly reduces moral balancing is the preclusion of social recognition. This entails that doing things in private seems to reflect differently on the agent, compared to doing them publicly. In a different line of research, a similar assumption is made. The literature on “costly signaling” (Griskevicius et al., 2010) assumes that people sometimes engage publicly in altruistic actions in order to increase their social status. This line of thought is based on theories of impression management (Tedeschi et al., 1971), which assume that individuals strive to appear consistent in the eyes of others in order to maintain their credibility and adhere to external prosocial norms. In their application of impression management theory, Griskevicius et al. (2010) found that activating status motives led people to choose more (altruistic) green products over more luxurious non-green products when shopping in public, but not in private. By showing their willingness and ability to incur costs for others in public, people seem to send a signal of superior status. This, in turn, is an evolutionary advantage (Griskevicius et al., 2010). One could speculate that privacy precludes people from sending costly signals, so they keep on striving until their behavior has been recognized. A similar argument has been made by Brick et al. (2017), who hypothesized that a valued social identity will more strongly drive identity-associated behavior when behavior is visible to others, because these actions underscore the individual’s reputation. Looking at our own data, an impression management account does appear somewhat congruent with the general nature of the effects we found, but it also contradicts the specific pattern that we found. Specifically, congruent with our data, an impression management account would emphasize the self-serving nature of prosocial striving. In other words, at least some people seem to perceive virtuous behavior as a costly resource that they need to limit and control - in particular, when their virtuousness has already been observed. From this perspective, virtuousness is a somewhat luxurious behavior that people engage in, but not infinitely, as it bears some risk of being exploited. However, in contradiction to our data, an impression management account would predict consistent (highlighting) and not inconsistent (balancing) prosocial striving under social recognition. Thus, it cannot explain our data the way that self-completion theory can.

### Limitations and Strengths of Both Studies

A somewhat natural limitation of our study is that our definition of prosocial vs. self-serving behavior may limit the scope of comparability with other results. Naturally, one has to define what prosocial behavior is and operationalize the variables accordingly. We defined prosocial behavior as a behavior that provides benefits for others, the environment, or society as a whole (cf. Schwartz, 1992, 2007). Correspondingly, we defined a behavior as self-serving when it aimed at one’s own benefit, neglecting the benefit of others. Following this definition, we cannot assume that all other studies which investigate moral balancing effects are fully comparable to our data. Previously published reviews (Effron and Monin, 2010; Blanken et al., 2015) demonstrate the wide range of definitions and operationalizations used in this field of study. While there is some evidence that balancing effects may be stronger (weaker) when different types of behavior represent similar (different) domains (Chatelain et al., 2018), the large body of evidence shows that balancing effects are not specific to one ethical domain but occur across domains - most likely based on similar, if not the same, mechanisms. Despite this, we are careful to generalize our results to other domains or argue that balancing effects in general would be dependent on social recognition. Nevertheless, we are convinced that it is important to be aware of the possible influence of social recognition in different paradigms and situations.

Finally, one potential limitation of our studies is that we collected our data in the laboratory. We did not test our hypotheses in the field. The subject of study - social recognition - calls for applied and naturalistic settings. It would be interesting to see whether the observed effects can be replicated under field conditions. On the positive side, we would argue that we made an effort to operationalize experiments in which we observed real behavior and assured real consequences from participant behavior (externalities for the university or charity; monetary or time budget consequences for the participants), which is quite rare in most of the literature on balancing phenomena. For instance, in Study 2, all donations resulted in actual payments to UNICEF and participants received donation receipts. We also analyzed our (pooled) data carefully using adequate and rather conservative non-parametric tests. In sum, our main results showed valid and reliable and, in our view, represent evidence for the relevance of Gollwitzer’s self-completion theory within moral balancing research.

### Future Research

We advocate that more replications are necessary to confirm our results. In order to assess the reliability of these results, independent replications are necessary. In addition, we propose that controlling for social recognition across a wider range of paradigms would be helpful to understand its impact better and more generally. Thus, a number of studies already published on moral balancing and highlighting implicitly allow for social recognition (see Blanken et al., 2015 for examples) without addressing it. We also second Blanken et al. (2015) in their call for including more participants in future studies, assuring sufficient power. We have tried to overcome this problem in Study 2, and partly succeeded. Finally, we think that investigating moderators of moral balancing in interaction with social recognition is a promising avenue for future studies. Some of the moderators identified by other researchers can be interpreted in the context of social recognition. For instance, Gneezy et al. (2012), as well as Clot et al. (2013), argue that receiving a financial reward for prosocial behavior changes subsequent prosocial striving. Social recognition could be factoring into this dynamic because, just like money, it turns a behavior into a “costly signal.” To conclude, our research shows that social recognition should definitely be considered in this area of research.

## Data Availability Statement

The datasets presented in this study can be found in online repositories. The names of the repository/repositories and accession number(s) can be found here: Open Science Framework, https://osf.io/23snv/files/.

## Ethics Statement

Ethical review and approval was not required for the study on human participants in accordance with the local legislation and institutional requirements. The patients/participants provided their written informed consent to participate in this study.

## Author Contributions

Both authors contributed to the study conception and design. MS and GW contributed to the material preparation and analysis and the data collection. MS wrote the first draft of the manuscript. Both authors commented on previous versions of the manuscript and read and approved the final manuscript.

## Conflict of Interest

The authors declare that the research was conducted in the absence of any commercial or financial relationships that could be construed as a potential conflict of interest.

## Footnotes

1 The terms *licensing* and *balancing* are often used interchangeably in the literature (Mullen and Monin, 2016). While *licensing* seems to be used solely in the context of ethical paradigms, *balancing* is used more generically to describe goal-regulation dynamics (Koo and Fishbach, 2008; Fishbach et al., 2009). We decided to use *balancing* (and only refer to *licensing* in exact quotes) in order to incorporate the perspective of non-ethical contexts. In our opinion, the most valuable theoretical frameworks such as self-completion theory or goal regulation theory also incorporate non-ethical contexts.

2 In some self-completion paradigms, the investigated motives are much more uncommon (“becoming a successful lawyer”), so the authors made an effort to preselect participants by their goals (see Gollwitzer et al., 2009, for example).

3 Due to a failure in the electronic data management base of the department, we could not retrieve the information on the age of 12 participants. Thus, the mean and standard deviation presented in the text reflects a sample of 112 participants.

4 For theoretical reasons, those participants should have been removed from the subsequent analyses. However, because the donation was completely anonymous in the condition without social recognition, in this condition we cannot reproduce in which cubicle the participants took their decisions. We therefore included all participants in our analyses.
